# EFFECTS OF A THEORY-ORIENTED SOCIAL FACILITATION INTERVENTION ON SOCIAL ISOLATION: A RANDOMIZED CONTROLLED TRIAL

**DOI:** 10.1093/geroni/igae098.3394

**Published:** 2024-12-31

**Authors:** Huimin Xiao, Huangqin Liu, Qiyuan Huang

**Affiliations:** Fujian Medical University, Fuzhou, Fujian, China (People’s Republic); Fujian Medical University, Fuzhou, Fujian, China (People’s Republic); Fujian Medical University, Fuzhou, Fujian, China (People’s Republic)

## Abstract

Transitioning to a nursing home represents significant life changes, which often result in a significant reduction and potential dissolution of pre-existing emotional bonds with family, friends, and social networks. Social facilitation is essential to help nursing home residents adapt a new life in nursing home. Thus, this study aimed to develop and examine the effects of the social facilitation program based on the cognitive-behavioral model of social isolation (SFCBSI), on social isolation, subjective well-being, cognitive tendencies, psychological adjustment, self-efficacy, and social support of nursing home residents. A randomized, controlled, assessor-blinded, parallel-arm trial was conducted in two nursing homes Fujian, Southeast of China from September 2023 to February 2024. 180 participants were randomly assigned to the intervention group (n = 90) and the control group (n = 90). The control group received routine institutional care. The intervention group received the SFCBSI program involving cognitive reconstruction, emotional management, and behavioral change, which was implemented by group for 4 weeks. Participants were assessed using the Cognitive Appraisal Orientation Test, Nursing Home Adjustment Scale, General Selfefficacy Scale, Perceived Social Support Scale, Social Isolation Scale and the Memorial University of Newfoundland Scale of Happiness Survey. Outcomes were evaluated at baseline and immediately after the intervention. The intervention participants showed significant differences in social isolation, subjective well-being, cognitive tendencies, psychological adjustment, self-efficacy, and social support, compared to the control group (P < 0.001). It indicates that the SFCBSI could reduce social isolation and improve psychological well-being among nursing home residents.

